# The Cross-Sectional Area Ratio of Right-to-Left Portal Vein Predicts the Effect of Preoperative Right Portal Vein Embolization

**DOI:** 10.3390/medicina60071114

**Published:** 2024-07-09

**Authors:** Yeongsoo Jo, Hae Won Lee, Ho-Seong Han, Yoo-Seok Yoon, Jai Young Cho

**Affiliations:** 1 Department of Surgery, Ewha Womans University Seoul Hospital, Ewha Womans University College of Medicine, Seoul 07804, Republic of Korea; dudtn87411@gmail.com; 2 Department of Surgery, Seoul National University Bundang Hospital, Seoul National University College of Medicine, Seoul 13620, Republic of Korea; hanhs@snubh.org (H.-S.H.); arsyun@gmail.com (Y.-S.Y.); jychogs@gmail.com (J.Y.C.)

**Keywords:** right portal vein embolization, future remnant liver volume, hepatectomy

## Abstract

*Background and Objectives:* Preoperative right portal vein embolization (RPVE) is often attempted before right hepatectomy for liver tumors to increase the future remnant liver volume (FRLV). Although many factors affecting FRLV have been discussed, few studies have focused on the ratio of the cross-sectional area of the right portal vein to that of the left portal vein (RPVA/LPVA). The aim of the present study was to evaluate the effect of RPVA/LPVA on predicting FRLV increase after RPVE. *Materials and Methods:* The data of 65 patients who had undergone RPVE to increase FRLV between 2004 and 2021 were investigated retrospectively. Using computed tomography scans, we measured the total liver volume (TLV), FRLV, the proportion of FRLV relative to TLV (FRLV%), the increase in FRLV% (ΔFRLV%), and RPVA/LPVA twice, immediately before and 2–3 weeks after RPVE; we analyzed the correlations among those variables, and determined prognostic factors for sufficient ΔFRLV%. *Results:* Fifty-four patients underwent hepatectomy. Based on the cut-off value of RPVA/LPVA, the patients were divided into low (RPVA/LPVA ≤ 1.20, N = 30) and high groups (RPVA/LPVA > 1.20, N = 35). The ΔFRLV% was significantly greater in the high group than in the low group (9.52% and 15.34%, respectively, *p* < 0.001). In a multivariable analysis, RPVA/LPVA (HR = 20.368, *p* < 0.001) was the most significant prognostic factor for sufficient ΔFRLV%. *Conclusions:* RPVE was more effective in patients with higher RPVA/LPVA, which is an easily accessible predictive factor for sufficient ΔFRLV%.

## 1. Introduction

The rate of liver cancer has been increasing over the past few decades [1]. Liver resection is the mainstay treatment for liver malignancy and can offer long-term survival for patients [2,3]. However, patients must fulfill certain criteria to be eligible for surgical resection, including having a sufficient future remnant liver volume (FRLV) [4]. FRLV is the percentage of the liver that will remain after surgical resection and is an independent and reliable predictor of posthepatectomy liver failure (PHLF) [4,5]. In other words, extensive liver resection poses a risk of PHLF if the remnant liver cannot meet the patient’s metabolic demand [6,7,8,9,10].

Since its introduction over 30 years ago, portal vein embolization (PVE) has become one of the treatment options used to reduce the risk of PHLF [11]. Embolization of the portal vein reduces portal vein flow to the targeted hemiliver with the tumor, which ultimately deprives the embolized hemiliver of the portal blood flow required to sustain its growth [12,13,14,15,16]. A physiological response to hypertrophy in the nonembolized hemiliver ensues. The unique dual vascular supply of the liver means that the risk of infarction after PVE is minimized [17].

In the last few decades, many studies have shown that PVE is safe and effective for downstaging liver cancer, optimizing FRLV, and ultimately increasing the number of patients suitable for major liver resection, allowing for the treatment of otherwise unresectable disease [18,19,20]. To achieve this benefit, it is important to measure FRLV precisely when selecting patients, and some researchers insist that manual measurement is mandatory [21,22]. However, volumetry measures FRLV after performing PVE and cannot predict it. Therefore, there is virtually no method to predict FRLV before performing PVE, despite its importance in establishing the risk of PHLF.

Because portal vein flow is a key factor involved in the increase in FRLV (ΔFRLV), we hypothesized that portal vein size, measured on computed tomography (CT) images, is correlated with the increase in the proportion of FRLV relative to TLV (FRLV%). If our hypothesis is positively correlated, the ratio of the cross-sectional area of the right portal vein to that of the left portal vein (RPVA/LPVA) can be used to predict the increase in FRLV% (ΔFRLV%), which would be feasible and very useful under many circumstances. Therefore, the aim of this study was to evaluate the effect of RPVA/LPVA on ΔFRLV% after right PVE (RPVE) and to analyze its utility for predicting ΔFRLV%.

## 2. Materials and Methods

### 2.1. Study Population

This was a single-center retrospective cohort study performed at Seoul National University Bundang Hospital, Seoul, Republic of Korea. A total of 78 patients who had undergone RPVE to increase FRLV between November 2004 and August 2021 were initially included in this study. Because of the long study period and the many surgeons involved, there were no definite inclusion criteria for preoperative RPVE, but FRLV, age, comorbidities, and liver function were considered. We excluded three patients who underwent left hepatectomy (N = 2), had not undergone postembolization CT (N = 1), and refused further treatment (N = 1). We also excluded patients with Child–Turcotte–Pugh (CTP) class B (N = 9) because they might have portal hypertension, which could influence hepatic regeneration. As a result, 65 patients were ultimately included. Fifty-four of these underwent right hepatectomy (RH), and we classified these patients into an RH group. Four patients did not undergo any surgery after embolization because of poor general condition or cancer progression, and the other seven patients underwent other types of surgery (palliative cholecystectomy, N = 4; open liver biopsy, N = 2; intraoperative radiofrequency ablation, N = 1) as palliative therapy because of gross peritoneal carcinomatosis, and we classified these eleven patients into a non-RH group. We planned to divide the patients into several groups according to RPVA/LPVA and analyze the statistical differences between them.

### 2.2. RPVA/LPVA

**Figure 1:**
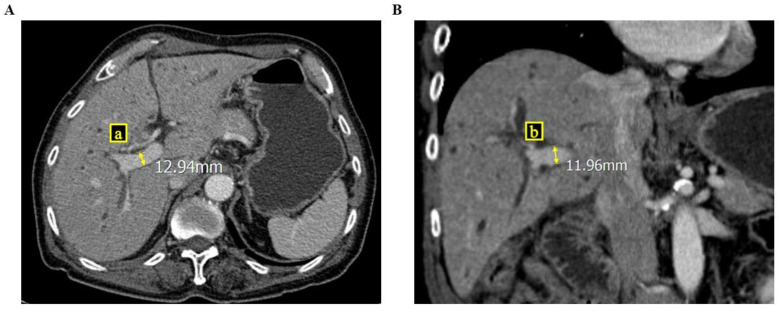
These figures show how to measure the cross-sectional area of the right portal vein via computed tomography ((**A**) cross section view; (**B**) coronary section view), where a and b are the semi-major and minor axes of the right portal vein.

**Figure 2:**
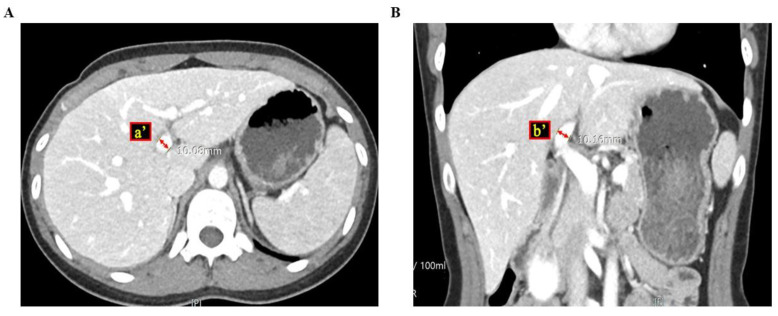
These figures show how to measure the cross-sectional area of the left portal vein via computed tomography ((**A**) cross section view; (**B**) coronary section view), where a’ and b’ are the semi-major and minor axes of the left portal vein.

To determine the ratio of the cross-sectional areas of the portal veins, we reviewed all CT images of the patients. Because blood vessels, including the portal vein, are cylindrical, the equation of the ratio of the cross-sectional areas was RPVA/LPVA = (π × a × b)/(π × a’ × b’), where a and b are the semi-major and minor axes of the right portal vein, respectively, and a’ and b’ are the semi-major and minor axes of the left portal vein, respectively [Figure 1 and Figure 2]. Parameters a and b were measured at a point within 5 mm of the bifurcation of the right and left portal veins. If there was any anatomical variation in the portal veins (e.g., trifurcation, or if the right posterior portal vein was the first branch of the main portal vein), we measured the axes on each branch and summed the areas to calculate the areas of the right, and the left portal vein was measured after the last branch of the right portal vein.

### 2.3. Statistical Analysis

All data were analyzed using SPSS version 20.0 for Windows (SPSS, Chicago, IL, USA) and R version 4.3.2 (R Development Core Team, Vienna, Austria, http://www.R-project.org). Continuous variables were compared with Student’s *t*-test. Categorical variables were compared with a χ^2^ test, linear-by-linear association, or Fisher’s exact test. We also performed univariate and multivariable analyses using logistic regression to identify the independent prognostic factors for ΔFRLV%. Furthermore, we used a recursive partitioning procedure to calculate the appropriate cutting point for classification. We also used a recursive partitioning procedure or tree classification algorithm in R to identify optimal cutting points for each marker [23]. In the multivariable analysis, we used all significant variables from the univariate analysis. In all tests, a *p* value of <0.05 was deemed significant.

## 3. Results

### 3.1. Baseline Characteristics of the Patients

We determined the cut-off value for RPVA/LPVA with the maximal χ^2^ method to define the largest ΔFRLV% permissible and divided the patients into two groups according to the cut-off value: the lower group (RPVA/LPVA ≤ 1.2) and the higher group (RPVA/LPVA > 1.2). Table 1 compares the lower (N = 30) and higher (N = 35) groups. No variable differed significantly between the two groups.

We divided the patients into two further groups according to whether they underwent RH (N = 54) or were non-RH (N = 11). Table 2 shows that there were significant differences between these groups in total bilirubin (0.91 and 1.39 mg/dL, respectively, *p* = 0.049) and INR (1.06 and 1.14, respectively, *p* = 0.044). These values did not differ significantly in the analysis according to RPVA/LPVA (Table 1).

### 3.2. Procedure-Related Characteristics of the Patients

Table 3 shows the comparisons between the low RPVA/LPVA and high RPVA/LPVA groups. The following parameters differed significantly between the low and high RPVA/LPVA groups: pre-FRLV% (42.47% and 35.49%, respectively, *p* < 0.001), the ratio of right and left hemiliver volume (RHLV/LHLV) before RPVE (pre-RHLV/LHLV; 1.43 and 1.94, respectively, *p* < 0.001), RPVA (25.12π and 34.39π mm^2^, respectively, *p* < 0.001), LPVA (27.83π and 19.91π mm^2^, respectively, *p* < 0.001), ΔFRLV% (9.52% and 15.34%, respectively, *p* < 0.001), and the RH rate (73.3% and 91.4%, respectively, *p* = 0.049).

Table 4 shows that ΔFRLV% was higher in the RH group than in the non-RH group (13.14% and 9.28%, respectively, *p* = 0.037), whereas pre-FRLV%, RPVA, LPVA, and RPVA/LPVA did not differ significantly between the two groups.

### 3.3. Surgical Outcomes

We performed a subgroup analysis of the RH group according to RPVA/LPVA; in the low RPVA/LPVA group, RPVA/LPVA was ≤1.2, and in the high-RPVA/LPVA group, RPVA/LPVA was >1.2, as in previous analyses. The results are shown in Table 5. The R0 resection rate was significantly greater in the high-RPVA/LPVA group than in the low-RPVA/LPVA group (84.8% and 53.8%, respectively, *p* = 0.009). However, there was no additional parameter that differed statistically significantly between the two groups in the analysis.

### 3.4. Prognostic Factors for Sufficient ΔFRLV%

We calculated the sufficient ΔFRLV% based on whether RH was performed or not because surgical resection is one of the definite treatment options for liver cancer. Using the recursive partitioning procedure, we found that the sufficient ΔFRLV% was 10%, an appropriate cutting point. In the univariate analysis, five factors were associated with sufficient ΔFRLV%: liver cirrhosis on radiological findings (hazard ratio [HR], 0.195; 95% confidence interval [CI], 0.041–0.923; *p* = 0.039), INR > 1.1 (HR, 0.287; 95% CI, 0.094–0.873; *p* = 0.028), HCC (HR, 0.219; 95% CI, 0.069–0.697; *p* = 0.010), adenocarcinoma (HR, 4.950; 95% CI, 1.569–15.618; *p* = 0.006), and RPVA/LPVA > 1.2 (HR, 21.577; 95% CI, 4.358–106.824; *p* < 0.001). In the multivariable analysis, HCC (HR, 0.185; 95% CI, 0.041–0.828; *p* = 0.027) and RPVA/LPVA > 1.2 (HR, 23.771 95% CI, 4.319–130.811; *p* < 0.001) were associated with sufficient ΔFRLV% (Table 6).

## 4. Discussion

The purpose of this study was to evaluate the effect of RPVA/LPVA on ΔFRLV% before RPVE, and it showed significant results. We identified RPVA/LPVA as a powerful prognostic factor for determining sufficient ΔFRLV%, especially when RPVA/LPVA was >1.2. This implies that the higher RHLV/LHLV% is in a patient before RPVE, the larger the regeneration volume of the left hemiliver after RPVE, which is probably because RPVA/LPVA reflects RHLV/LHLV%. As shown in Table 3, there was a significant difference in RHLV/LHLV% before RPVE (pre-RHLV/LHLV%) between the low and high RPVA/LPVA groups (1.43 and 1.94, respectively, *p* < 0.001). This result supports the assumption that RPVA/LPVA is correlated with pre-RHLV/LHLV. Certainly, measuring pre-RHLV/LHLV might be helpful for predicting ΔFRLV%, but it requires volumetry equipment and takes much longer than simply calculating RPVA/LPVA. Therefore, the measurement of RPVA/LPVA should be useful for selecting patients suitable for RPVE, as it can be calculated rapidly without volumetry and is a much easier method that involves interpreting CT images.

However, the present study had several limitations. First, it was a retrospective single-center study, which may limit the generalizability of the findings. Second, the disease groups were heterogeneous, and no disease-specific subgroup analysis was performed, potentially confounding the results. Third, the sample size used to determine the cut-off point for ΔFRLV% for classification was relatively small, which could affect the robustness of our conclusions. Fourth, we did not measure the velocity in both portal veins, so it was not possible to confirm whether these velocities were fixed or identical in the right and left portal veins. Fifth, we excluded patients with CTP-B due to concerns that this could influence liver regeneration after RPVE. Nevertheless, many patients with liver malignancies, particularly HCC, have liver cirrhosis. The prognostic role of liver function in hepatocellular carcinoma (HCC) treatment was noted in a comprehensive review [24], and liver resection is generally not recommended for patients with deteriorated liver function (e.g., CTP-B or C) due to the high risk of postoperative complications, including PHLF. To increase the rate of curative resections, further research is needed to investigate the effects of preoperative PVE in patients with borderline liver function and liver malignancy. These issues warrant further investigation in future studies.

## 5. Conclusions

Clearly, there are many different types of liver volumetry programs, so it is not easy to say that RPVA/LPVA measurement is always more useful than such programs. However, we believe that identifying the association between RPVA/LPVA and ΔFRLV% is an important basis for developing an alternative method that could avoid laborious volumetry. To the best of our knowledge, this is the first study to identify the correlation between portal vein size and ΔFRLV%. Based on our results, more studies on this issue are warranted.

## Figures and Tables

**Table 1:** Baseline characteristics comparing the RPVA/LPVA ≤1.2 and >1.2 groups.

	≤1.2 (N = 30)	>1.2 (N = 35)	*p* Value
Age (mean)	64.57	62.97	0.599
Age ≥ 65	18 (60.0%)	15 (42.9%)	0.168
Sex (male)	7 (23.3%)	10 (28.6%)	0.632
BMI (mean)	23.73	23.4	0.712
LC (radiology)	6 (20.0%)	2 (5.7%)	0.085
HBV (+)	6 (20.0%)	6 (17.1%)	0.767
HCV (+)	3 (10.0%)	0 (0.0%)	0.093
Serum creatinine (mean)	0.81	0.81	0.983
Serum AST (mean)	48.4	44.1	0.462
Serum ALT (mean)	49.9	47.9	0.777
Serum albumin (mean)	3.76	3.84	0.435
Serum TB (mean)	1.06	0.93	0.302
Serum platelet (mean)	262.33	247.17	0.578
INR (mean)	1.10	1.04	0.057
Ascites	2 (6.7%)	0 (0.0%)	0.209
Disease			
HCC	11 (36.7%)	8 (22.9%)	0.222
pCCA	13 (43.3%)	16 (45.7%)	0.847
GBC	2 (6.7%)	3 (8.6%)	0.574
CRLM	2 (6.7%)	7 (20.0%)	0.116
Others	2 (6.7%)	1 (2.9%)	0.442
Adenocarcinoma	17 (56.7%)	27 (77.1%)	0.078

RPVA/LPVA—the ratio of the cross-sectional area of the right portal vein to that of the left portal vein, BMI—body mass index, LC—liver cirrhosis, HBV—hepatitis B virus, HCV—hepatitis C virus, AST—aspartate transaminase, ALT—alanine transaminase, TB—total bilirubin, INR—international normalized ratio, HCC—hepatocellular carcinoma, pCCA—proximal cholangiocarcinoma, GBC—gallbladder cancer, CRLM—colorectal liver metastasis.

**Table 2:** Baseline characteristics comparing the RH and the non-RH groups.

	RH (N = 54)	Non-RH (N = 11)	*p* Value
Age (mean)	63.93	62.64	0.749
Age ≥ 65	27 (50.0%)	6 (54.5%)	0.783
Sex (male)	16 (29.6%)	1 (9.1%)	0.149
BMI (mean)	23.89	21.89	0.092
LC (radiology)	6 (11.1%)	2 (18.2%)	0.408
HBV (+)	9 (16.7%)	3 (27.3%)	0.327
HCV (+)	3 (5.6%)	0 (0.0%)	0.568
Serum creatinine (mean)	0.79	0.92	0.072
Serum AST (mean)	46.00	46.55	0.944
Serum ALT (mean)	49.02	47.73	0.893
Serum albumin (mean)	3.81	3.76	0.750
**Serum TB (mean)**	**0.91**	**1.39**	**0.049**
Serum platelet (mean)	250.04	274.45	0.500
**INR (mean)**	**1.06**	**1.14**	**0.044**
Ascites	1 (1.9%)	1 (9.1%)	0.312
Disease			
HCC	15 (27.8%)	4 (36.4%)	0.406
pCCA	24 (44.4%)	5 (45.5%)	0.603
GBC	3 (5.6%)	2 (18.2%)	0.196
CRLM	9 (16.7%)	0 (0.0%)	0.166
Others	3 (5.6%)	0 (0.0%)	0.568
Adenocarcinoma	37 (68.5%)	7 (63.6%)	0.504

RH—right hepatectomy, BMI—body mass index, LC—liver cirrhosis, HBV—hepatitis B virus, HCV—hepatitis C virus, AST—aspartate transaminase, ALT—alanine transaminase, TB—total bilirubin, INR—international normalized ratio, HCC—hepatocellular carcinoma, pCCA—proximal cholangiocarcinoma, GBC—gallbladder cancer, CRLM—colorectal liver metastasis.

**Table 3:** Comparison between the RPVA/LPVA ≤ 1.2 and >1.2 groups.

	≤1.2 (N = 30)	>1.2 (N = 35)	*p* Value
**Pre-FRLV% (mean)**	**42.47%**	**35.49%**	**<0.001**
**Pre-RHLV/LHLV (mean)**	**1.43**	**1.93**	**<0.001**
**RPVA (π mm** **^2^** **; mean)**	**25.12**	**34.39**	**<0.001**
**LPVA (π mm** **^2^** **; mean)**	**27.83**	**19.91**	**<0.001**
Post-FRLV% (mean)	51.99%	50.83%	0.573
**ΔFRLV% (mean)**	**9.52%**	**15.34%**	**<0.001**
**Operation type**			**0.049**
**RH**	**22 (73.3%)**	**32 (91.4%)**	
**Non-RH**	**8 (26.7%)**	**3 (8.6%)**	

RPVA/LPVA—the ratio of the cross-sectional area of the right portal vein to that of the left portal vein, FRLV—future remnant liver volume, FRLV%—the proportion of FRLV relative to total liver volume, Pre-FRLV%—FRLV% before right portal vein embolization, Pre-RHLV/LHLV%—the ratio of right and left hemiliver volume, RPVA—right portal vein area, LPVA—left portal vein area, PVA—polyvinyl alcohol, Post-FRLV%—FRLV% after right portal vein embolization, ΔFRLV%—increase in FRLV%, RH—right hepatectomy.

**Table 4:** Comparison between the RH and the non-RH groups.

	RH (N = 54)	Non-RH (N = 11)	*p* Value
Pre-FRLV% (mean)	38.71%	38.72%	0.996
RPVA (π mm^2^; mean)	10.84	10.36	0.428
LPVA (π mm^2^; mean)	9.36	9.63	0.610
RPVA/LPVA (mean)	1.41	1.33	0.728
Post-FRLV% (mean)	51.84%	49.00%	0.295
**ΔFRLV% (mean)**	**13.14%**	**9.28%**	**0.037**

RH—right hepatectomy, FRLV—future remnant liver volume, FRLV%—the proportion of FRLV relative to total liver volume, Pre-FRLV%—FRLV% before right portal vein embolization, RPVA—right portal vein area, LPVA—left portal vein area, RPVA/LPVA—the ratio of the cross-sectional area of the right portal vein to that of the left portal vein, PVA—polyvinyl alcohol, Post-FRLV%—FRLV% after right portal vein embolization, ΔFRLV%—increase in FRLV%.

**Table 5:** Surgical outcomes of RH according to RPVA/LPVA > 1.2.

	≤1.2 (N = 25)	>1.2 (N = 32)	*p* Value
Pringle maneuver	4 (16.0%)	3 (9.4%)	0.360
Pringle time	3.52	3.91	0.898
Major vessel reconstruction	2 (8.0%)	5 (15.6%)	0.327
Operation time	304.26	299.47	0.869
Estimated blood loss	826.91	725.76	0.626
Posthepatectomy liver failure	1 (4.5%)	1 (3.1%)	0.653
CDC ≥ IIIa	14 (51.9%)	12 (35.3%)	0.194
**R0 resection**	**14 (53.8%)**	**28 (84.8%)**	**0.009**
Postoperative hospital stay	16.58	16.56	0.996

RH—right hepatectomy, RPVA/LPVA—the ratio of the cross-sectional area of the right portal vein to that of the left portal vein, CDC—Clavien-Dino classification.

**Table 6:** Prognostic factors for ΔFRLV% ≥ 10%.

Variables	Univariate Analysis			Multivariable Analysis		
	HR	95% CI	*p* Value	HR	95% CI	*p* Value
Age ≥ 65	0.667	0.227–1.961	0.461			
Sex (male)	1.012	0.300–3.410	0.985			
BMI	0.949	0.818–1.101	0.491			
LC (radiology)	0.195	0.041–0.923	0.039	1.193	0.116–12.219	0.882
HBV (+)	0.503	0.137–1.844	0.300			
HCV (+)	0.189	0.016–2.221	0.185			
Serum creatinine (mean)	1.437	0.113–18.302	0.780			
Serum AST (mean)	0.992	0.970–1.014	0.489			
Serum ALT (mean)	1.007	0.987–1.027	0.503			
Serum albumin (mean)	1.196	0.323–4.423	0.789			
Serum TB (mean)	1.381	0.479–3.981	0.551			
Serum platelet (mean)	0.999	0.994–1.004	0.643			
INR > 1.1	0.287	0.094–0.873	0.028	0.432	0.104–1.791	0.248
Ascites	0.400	0.024–6.746	0.525			
**Disease**						
**HCC**	**0.219**	**0.069–0.697**	**0.010**	**0.185**	**0.041–0.828**	**0.027**
pCCA	1.571	0.525–4.707	0.419			
CRLM	3.789	0.440–32.635	0.225			
Others	0.818	0.070–9.599	0.873			
Adenocarcinoma	4.950	1.569–15.618	0.006	1.992	0.100–39.709	0.652
**RPVA/LPVA > 1.2**	**21.577**	**4.358–106.824**	**<0.001**	**23.771**	**4.319–130.811**	**<0.001**

FRLV—future remnant liver volume, FRLV%—the proportion of FRLV relative to total liver volume, Pre-FRLV%—FRLV% before right portal vein embolization, BMI—body mass index, LC—liver cirrhosis, HBV—hepatitis B virus, HCV—hepatitis C virus, AST—aspartate transaminase, ALT—alanine transaminase, TB—total bilirubin, INR—international normalized ratio, CTP—Child-Turcotte-Pugh, HCC—hepatocellular carcinoma, pCCA—proximal cholangiocarcinoma, GBC—gallbladder cancer, CRLM—colorectal liver metastasis, RPVA/LPVA—the ratio of the cross-sectional area of the right portal vein to that of the left portal vein.

## Data Availability

The datasets presented in this article are not readily available because of patients’ privacy and legal/ethical issues.
